# Ultrasensitive detection of vital biomolecules based on a multi-purpose BioMEMS for Point of care testing: digoxin measurement as a case study

**DOI:** 10.1038/s41598-024-51864-4

**Published:** 2024-01-18

**Authors:** Fahimeh Marvi, Kian Jafari, Mahmoud Shahabadi, Maryam Tabarzad, Fatemeh Ghorbani-Bidkorpeh, Taha Azad

**Affiliations:** 1 CenBRAIN Neurotech Center of Excellence, School of Engineering, Westlake University, Hangzhou, China; 2 Mechanical Engineering Department, Faculty of Engineering, Université de Sherbrooke, 2500 Boul. de l’Université, Sherbrooke, QC Canada; 3 Interdisciplinary Institute for Technological Innovation (3IT), Université de Sherbrooke (UdeS), Quebec, J1K 2R1 Canada; 4 School of Electrical and Computer Engineering, College of Engineering, University of Tehran, Tehran, Iran; 5 Protein Technology Research Center, Shahid Beheshti University of Medical Sciences, Tehran, Iran; 6 Department of Pharmaceutics and Pharmaceutical Nanotechnology, School of Pharmacy, Shahid Beheshti University of Medical Sciences, Tehran, Iran; 7 Faculty of Medicine and Health Sciences, Department of Microbiology and Infectious Diseases, Université de Sherbrooke, Sherbrooke, QC J1E 4K8 Canada; 8 Centre de Recherche du CHUS, Sherbrooke, QC J1H 5N4 Canada

**Keywords:** Electrical and electronic engineering, Mechanical engineering

## Abstract

Rapid and label-free detection of very low concentrations of biomarkers in disease diagnosis or therapeutic drug monitoring is essential to prevent disease progression in Point of Care Testing. For this purpose, we propose a multi-purpose optical Bio-Micro-Electro-Mechanical-System (BioMEMS) sensing platform which can precisely measure very small changes of biomolecules concentrations in plasma-like buffer samples. This is realized by the development of an interferometric detection method on highly sensitive MEMS transducers (cantilevers). This approach facilitates the precise analysis of the obtained results to determine the analyte type and its concentrations. Furthermore, the proposed multi-purpose platform can be used for a wide range of biological assessments in various concentration levels by the use of an appropriate bioreceptor and the control of its coating density on the cantilever surface. In this study, the present system is prepared for the identification of digoxin medication in its therapeutic window for therapeutic drug monitoring as a case study. The experimental results represent the repeatability and stability of the proposed device as well as its capability to detect the analytes in less than eight minutes for all samples. In addition, according to the experiments carried out for very low concentrations of digoxin in plasma-like buffer, the detection limit of LOD = 300 fM and the maximum sensitivity of S = 5.5 × 10^12^ AU/M are achieved for the implemented biosensor. The present ultrasensitive multi-purpose BioMEMS sensor can be a fully-integrated, cost-effective device to precisely analyze various biomarker concentrations for various biomedical applications.

## Introduction

Nowadays, chronic diseases or noncommunicable diseases are one of the most important cause of death in the world (74% of all deaths [WHO, 2019]). Furthermore, the treatment or control of these diseases are very expensive compared to the early diagnosis and prevention processes^1,2^. Consequently, the development of high-performance and cost-effective transducers for label-free detection of specific biomarkers, particularly for chronic diseases, is incredibly appealing to improve the human life quality by controlling the disease progression and drug monitoring for dosage adjustment in point-of-care (POC) testing. This may also decrease the total health cost expenditures of the world-wide health systems^3–5^. In this context, biosensing technologies are good alternatives for time-consuming and expensive conventional methods of early diagnosis or therapeutic drug monitoring, performed based on immunoassay analyzers or mass spectrometers in the centralized laboratories^6–8^. In fact, these efficient bio-platforms can appropriately measure target biomolecules (and their concentrations in vital fluids) which are related to a specific disease (and its progress) for early diagnosis of that specific disease. Also, they can precisely measure the drug concentration in serum (or blood) samples at timed intervals in order to maintain the medication level in a targeted therapeutic range^9,10^.

On the other hand, several factors play key roles to realize an ultra-sensitive biosensing platform such as sensitive transducers and precise detection systems^11^. These factors are significant particularly where interactions between target bioreceptor-analyte should be precisely measured for an ultra-sensitive detection. Recently, several approaches have been implemented to improve these factors. For instance, mass-based microelectromechanical (dynamic) biosensors operations are relied on frequency changes of a cantilever caused by variations in biological masses of samples^12^. While, surface stress-based (static) biosensors rely on the static operation of a MEMS cantilever so that the cantilever displacement can determine biomarker concentrations^13,14^. However, there are still some challenges in the commercialization of such electromechanical biosensing platforms. The most of micro-resonators for mass-based biosensing suffer from Q-factor mitigation in a fluidic sample due to damping phenomenon^15,16^. In surface stress-based methods, the cantilever operation is critically dependent to the bioreceptor immobilization and its surface functionalization^13,14^. These structures also need a reliable and high-resolution detection system which can be integrated with biosensor in a small device to accurately detect variations in the M/NEMS cantilever position (≤ nm)^17–19^. Besides, several recent proposed methods are *ad-hoc* and cannot be generalized for various biosensing applications (e.g. glucose sensors^20^). To overcome some of these shortcomings, we present in this work an implementation of a multi-purpose biosensing platform based on an optical BioMEMS structure for ultra-sensitive detection of various biomarkers. The identification and measurement of digoxin, (well-known as a cardiac glycoside medication to treat or manage heart failure and atrial fibrillation), is also carried out as a case study. The importance of selecting digoxin as our case study for the present multi-purpose bio-platform is due to the fact that heart diseases such as heart failure and atrial fibrillation are major health problems affecting many people around the globe (16% of total deaths in recent years according to the WHO reports)^21,22^. These chronic diseases frequently coexist and may mutually enhance their progression processes and thus the risk of mortality (with an estimated total cost of treatment around $69.7 billion in USA by 2030)^23,24^. Although this medication is essential in heart rate control of patients with atrial fibrillation, its narrow therapeutic index limits the use of digoxin due to the possibility of its toxicity. This challenge can be resolved by a regular monitoring of serum concentrations to adjust digoxin dosage in the beginning of the medication usage^25–27^. However, the regular measurement and monitoring should be carried out by inexpensive and rapid approaches, in contrast to the existing conventional methods (i.e., chromatographic methods or immunoassays)^28^. This is exactly where the proposed multi-purpose bio-platform based on optical BioMEMS technology can effectively find its place to provide sensitive, inexpensive and rapid service of identification and monitoring to the public.

As a consequence, in the following, to provide an inexpensive device for ultra-sensitive detection of various biomarkers, an optical BioMEMS sensing platform and its numerical and analytical analysis have been proposed. Several experiments have been also carried out by the functionalized sensors for measurement of digoxin concentrations (as our case study) in order to present a good candidate for rapid and cost-effective therapeutic drug monitoring in POC applications. Furthermore, the biosensor operation was analyzed by coating different packing densities of bio-receptors on its surface. A comparative study has been further carried out to show the importance of the proposed BioMEMS platform in terms of its functional characteristics compared to several important approaches in the same field.

### Multi-purpose optical MEMS (MOM) bio-platform for ultra-sensitive detection of biomolecules

**Figure 1:**
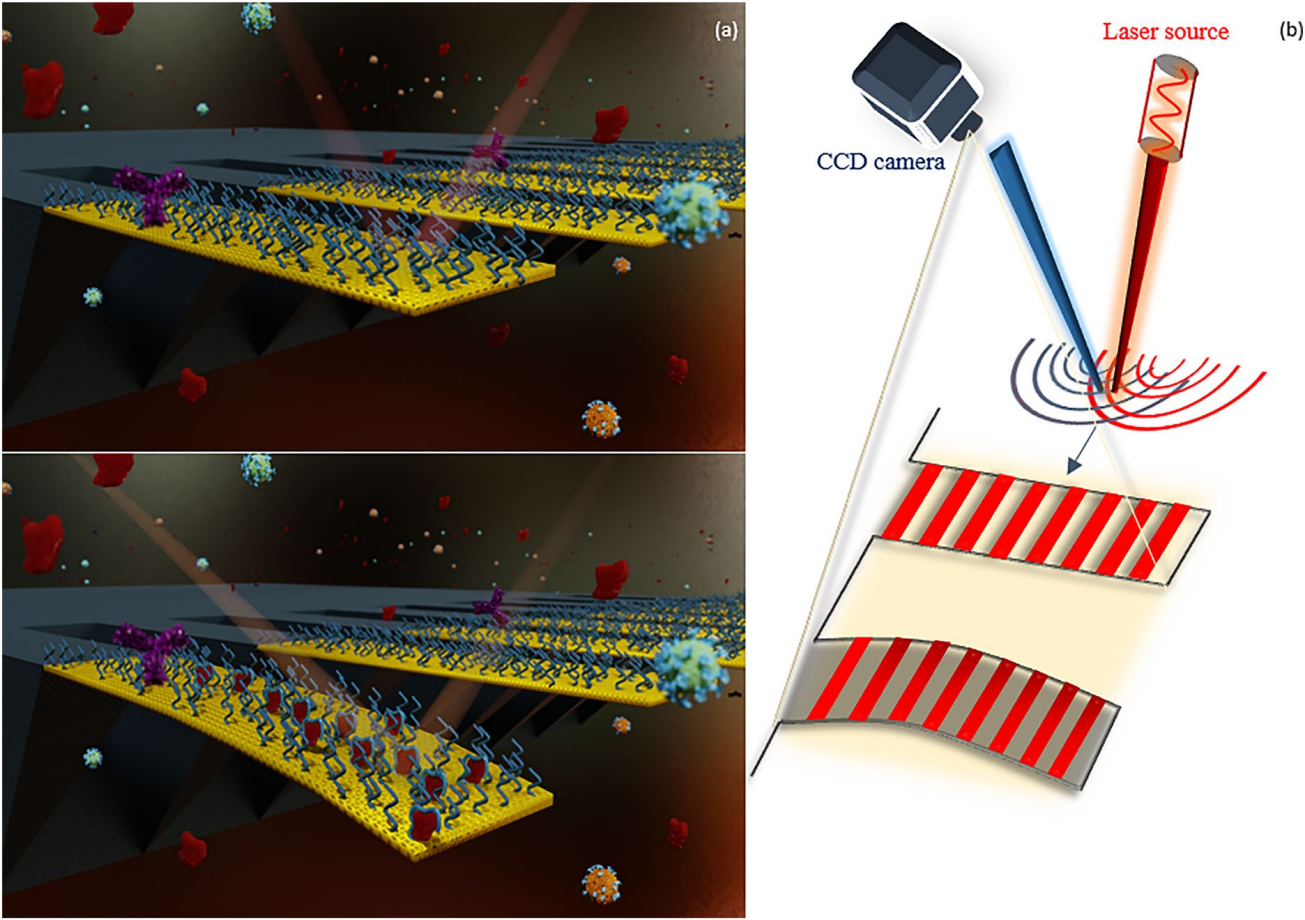
A schematic description of (**a**) the functionalized BioMEMS platform exposed to the target samples, (**b**) the MEMS transducer responses under an induced surface stress accompanied by the proposed optical measurement method to record this reaction.

**Figure 2:**
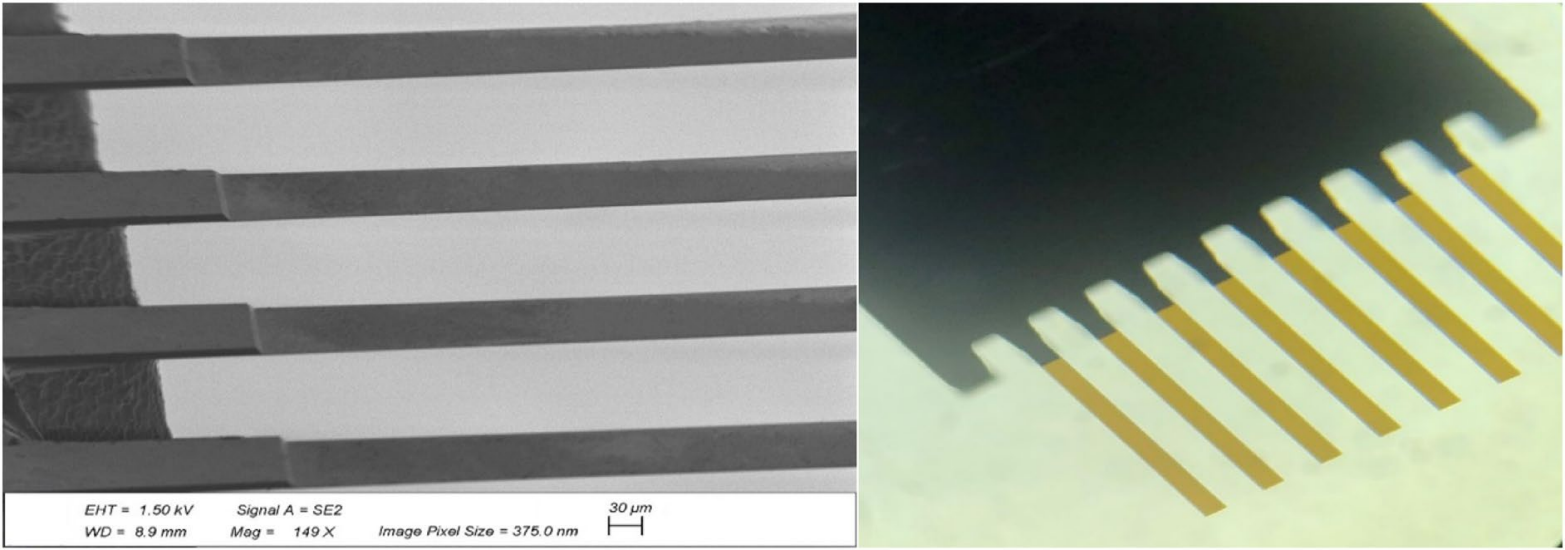
Scanning electron microscope (SEM) of the cantilevers (left) and a top view of the cantilevers (right).

**Figure 3:**
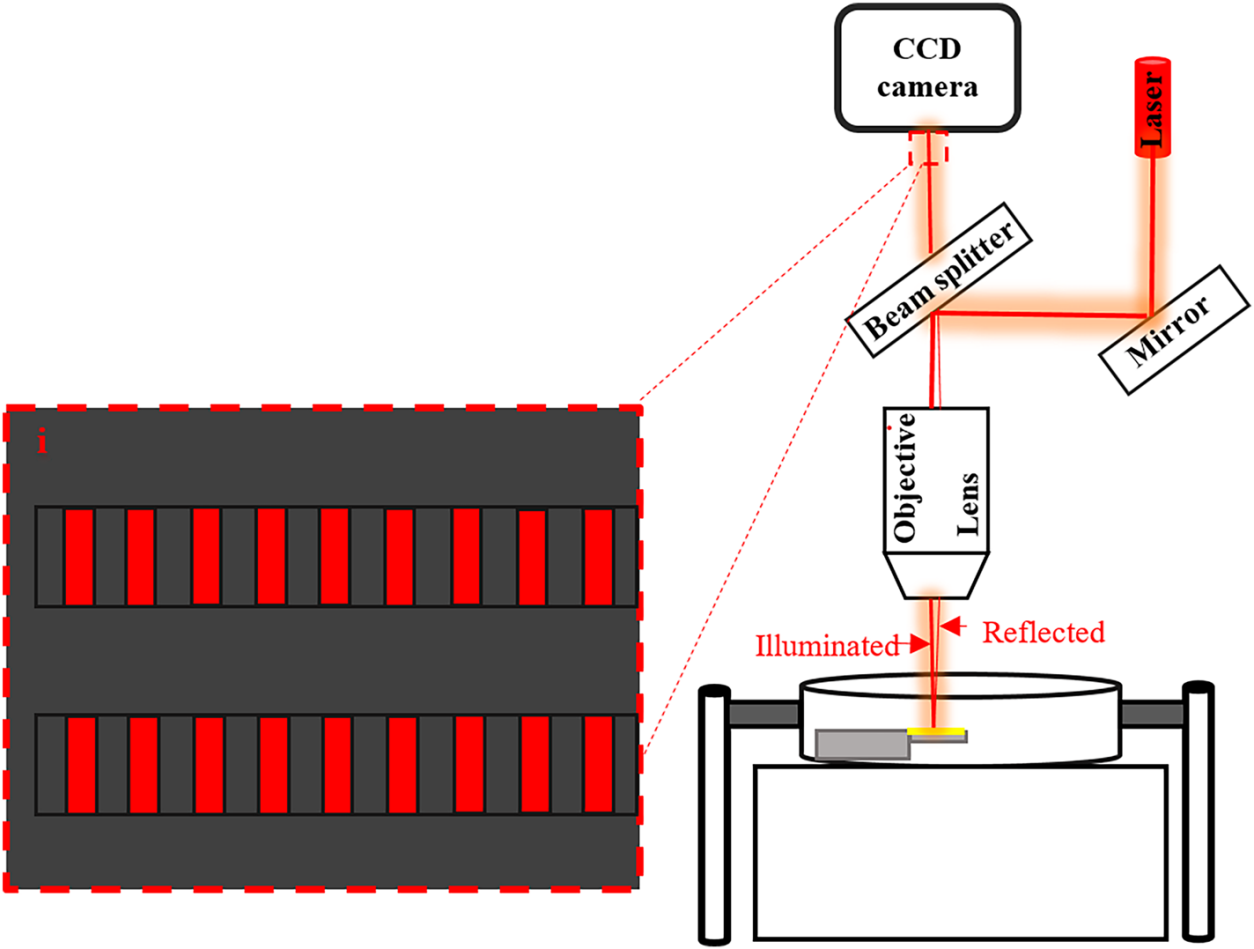
Schematic description of the proposed optical measurement setup. Note that part (i) illustrates interferometry pattern on the MEMS cantilevers captured by CCD camera.

Considering the current challenges and limitations mentioned earlier for dynamic and static-based MEMS biosensors, our BioMEMS platform can address such challenges for measuring biological quantities. The present biosensor is proposed based on a highly sensitive BioMEMS cantilever, which plays the role of a movable mirror in the presented tunable MEMS interferometer. Various changes in the position of the MEMS transducer induce significant alterations in the interference pattern of the optical detection system. Using a CCD camera and applying a designed image processing algorithm facilitate the data recording to precisely quantify the biological targets. This is realized by implementing the algorithm through taking several images from each sample during the tests, leading to obtaining multiple data in a short period of time and providing an average image. The subtraction of this average image from the reference image then results in eliminating any errors or noises induced by unwanted displacements, laser-induced heating effect, or changes in environmental conditions. Consequently, the proposed BioMEMS device can precisely detect very small displacements by basic optical elements without the need for complicated alignments or complex systems. Thus, it can be an appealing alternative for the time-consuming and expensive conventional methods used for early diagnosis and therapeutic drug monitoring, often relying on immunoassay analyzers or mass spectrometers in centralized laboratories. The schematic description of the proposed multi-purpose BioMEMS sensor is illustrated in Fig. 1. Silicon cantilevers fabricated on a substrate (Fig. 2) play the role of MEMS transducers, coated by a thin gold layer to facilitate the bio-functionalization procedure of the cantilevers and to increase light reflection. These cantilevers have been designed for a sensitive biological operation in the static mode. In fact, the sensing mechanism of the BioMEMS part is based on the released stresses on the cantilever surface which can deflect it up (or down). This surface stress is a collective phenomenon^29^ which is strongly dependent on the binging events (biological interactions) and on the amount of released energies. Since the biological reaction forces are usually in the scale of pico- or nano-newtons, a flexible MEMS transducer with a high sensitivity should be designed to be extremely sensitive in biosensing applications. Therefore, our cantilevers have been designed and implemented to satisfy the need for interacting with pico- or nano-newtons forces caused by biological reactions. The cantilever dimensions are $L = 1000\;\upmu {\text{m}},\;W = 90\;\upmu {\text{m}},\;t = 1\;\upmu {\text{m}}$ which result in a spring constant of $k = 0.0032\;{\text{N}}/{\text{m}}$ for each MEMS structure. This small stiffness of the implemented cantilevers causes large displacements even with very small changes of the induced surface stresses. After implementing the multi-purpose platform, a successful bio-functionalization process on the cantilever surfaces can enhance the sensor efficiency to convert biological interactions into mechanical signals. For this purpose, appropriate recognition elements with high specificity and high selectivity should be firstly selected based on the desired diagnostic or therapeutic aims and then continuously immobilized on the cantilever surfaces. In this step, the functionalized MEMS cantilevers should be equipped with a high resolution and cost-effective measurement system to be an appealing candidate for POC Testing. Thus, an ultra-sensitive optical detection structure is implemented for the proposed platform which records tiny changes of cantilevers. The setup is implemented as shown in Fig. 3 by an assembled microscope, taking the advantages of the optical interferometry technology (Fig. 1b).

As represented in Fig. 3, the red light of a conventional laser diode is reflected by a mirror to a beam splitter. A part of illuminated waves is transmitted through an objective lens to be focused as a spot light on the cantilever surfaces. This results in a light reflection from the Au coating that is collected by the objective lens and returned to the beam splitter. Because of the phase difference between these forward and backward waves, an interference pattern of electromagnetic waves thus appears on the cantilevers which is a periodic corrugated model, as typically shown in Figs. 1 and 3. Finally, a high-resolution charge-coupled device (CDD) (or a CCD camera) records the images of experimental measurements and sends them to a processor (i.e., a smartphone, a laptop or even on a cloud) for the image processing step.

**Figure 4:**
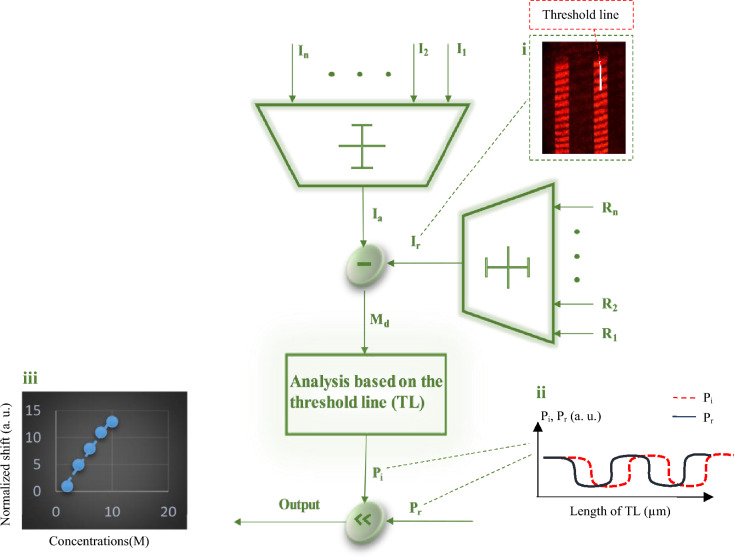
Schematic description of the image processing algorithm developed for the proposed UOM bio-platform. Note that (i) shows the single reference image $(I_{r} )$ with the corrugated optical pattern and the threshold line, (ii) represents profiles of the red pixel intensity $\left( {P_{i} } \right)$ and the reference profile $\left( {P_{r} } \right)$ and (iii) illustrates the calibration curve of the proposed BioMEMS sensor plotted by position shifts of the red pixel intensity profiles (due to various concentrations).

The proposed image processing algorithm, illustrated schematically in Fig. 4, determines the shifts of the pixel intensity profile and thus the changes in the cantilever position caused by the biological interactions between the specific bio-receptors and the target bio-molecules. Therefore, the algorithm can not only identify the target biomolecules (i.e., medication type, sort of disease) but also measure their concentrations (i.e., drug dosage, stage of progress or contamination) in the vital fluids. In this image processing approach (Fig. 4), all captured images of each experiment are given to the algorithm as the inputs $\left( {I_{1} , I_{2} , \ldots , I_{n} } \right)$. Also, the cantilever zero-position images captured in a buffer injection (without digoxin) are used as the reference images $\left( {R_{1} , R_{2} , \ldots , R_{n} } \right)$. In the first step, these various captured input images and references images are separately averaged to obtain respectively a single image (so-called the average image, $I_{a}$) and a single reference image, $I_{r}$. A differential single image $\left( {I_{d} } \right)$ is then generated by subtracting the reference image $(I_{r} )$ from the average image $(I_{a} )$. A hypothetical straight line (so-called “threshold line”) is defined perpendicular to the corrugated optical pattern (as shown in the reference image of Fig. 4).

Note that this initial position of the hypothetical threshold line (TL) should be kept unchanged for all the measurements so that one can report any change in the cantilever position induced under injection of various digoxin concentrations. Consequently, the 2 × 2 matrix $\left( {M_{d} } \right)$ of differential image is then analyzed based on the threshold line position so that one can obtain the red pixel intensity profile $\left( {P_{i} } \right)$ along the threshold line for each concentration (as can be seen in Fig. 4). According to the position shifts in the red pixel intensity profiles $\left( {P_{i} } \right)$ compared to the reference profile $\left( {P_{r} } \right)$, the calibration curve of the present biosensor can be obtained in order to measure the sensitivity and its detection limit.

It should be mentioned that the proposed approach can be used for various types of target bio-molecules and the algorithm can be applied for monitoring various types of medications in samples or for identification and concentration measurement of particular bio-molecules in vital fluids (i.e., blood, spinal fluid) to provide effective early diagnostic methods.

### Experimental implementation of the MOM bio-platform for Digoxin identification and measurement

**Figure 5:**
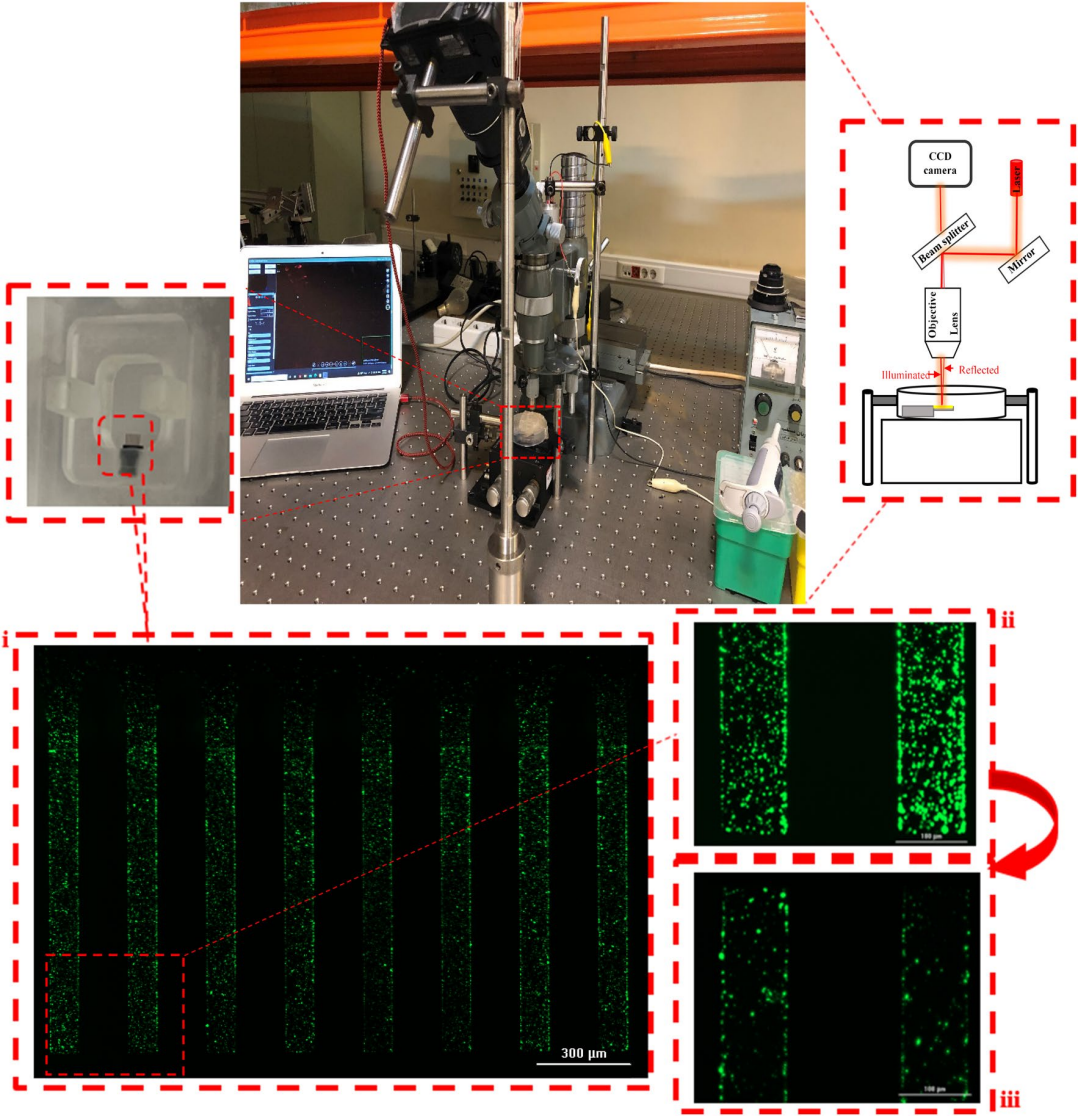
Experimental measurement setup implemented as a therapeutic drug monitoring device for identification and measurement of digoxin as a case study. Note that part (i) shows a fluorescent image of immobilizing the thiolated-aptamer bioreceptors on the biosensor surface with: (ii). A magnified image of the top sides of the MEMS cantilevers which are coated by a thin gold layer and (iii). A magnified image of th e other side of the silicon cantilevers.

In this Section, the proposed platform has been practically implemented to identify and measure the concentration of digoxin in serum as a therapeutic drug monitoring device. This experimental measurement setup is shown in Fig. 5.

#### MEMS transducer preparation setup

The transducers have been provided as eight silicon cantilevers with an Au coating for the immobilization of bioreceptors and optical detection of nanomechanical changes. To provide an appropriate test condition for this MEMS part, we fabricated a designed PMMA dish (plate) by CNC machining (4 mm resolution) consisting of a holder and a container in the millimeter scale. All biological procedures and optical tests reported in this manuscript have been performed on the proposed biosensing platform by fixing its plate on a base.

#### Bioreceptors immobilization procedure

In this case study, the fabricated MEMS cantilevers have been functionalized by synthesized anti-digoxin ssDNA aptamers [with the sequence of 5′-Thiol-C6-AGC GAG GGC GGT GTC CAA CAG CGG TTT TTT CAC GAG GTT GGC GGT GG-3′] (thanks to their better stability in biological fluids compared to the RNA ones) for therapeutic drug monitoring of digoxin. This procedure has been appropriately carried out by Self-Assembly Monolayer (SAM) based ligand immobilization of the thiol-modified aptamers on the surface of gold coated cantilevers. It should be mentioned that unlike other studies employing this aptamer, which necessitated the removal of proteins from real biological samples as a pretreatment before sensing digoxin^30^. our approach involved the implementation of a BioMEMS aptasensor for digoxin detection and quantification without the need for additional sample pretreatment. This is also essential for point-of-care testing devices that can be used independently by individuals, eliminating the need to refer to central laboratories for carrying out pre-treatments.

As already mentioned, surface stress generation is a collective phenomenon which is directly related to the surface coverage of bio-receptors on one side of the biosensor and the passivation of another side. Therefore, according to the importance of the surface stress generation in the mechanical displacement of a surface stress-based biosensor, a threshold coverage (with high continuity) of bio-recognition elements should be immobilized on the sensor surface to achieve a measurable static response. For this purpose, the biosensor surfaces have been exposed to the different packing densities of anti-digoxin aptamers concentrations in the ranges of $C_{p0} \sim0.5\;\upmu {\text{M}}$, $C_{p1} = 1\;\upmu {\text{M}}$ and $C_{p2} = 3\;\upmu {\text{M}}$ to prepare the cantilevers for various experiments. Prior to use, thiolated anti-digoxin aptamers have been activated by TCEP and then prepared in these three concentrations. In each surface functionalization, the fabricated PMMA plate of the MOM platform is firstly washed by an ultrasonic cleaner for five minutes. In addition, these chambers have been exposed to bovine serum albumin (BSA) for one hour to block its excess binding sites and prevent the physical adsorption. It is then washed thoroughly by excess deionized water to remove free BSA. Finally, the biosensor is washed three times by absolute-ethanol (provided from Sigma-Aldrich) and is incubated in $500\;\upmu {\text{L}}$ of the specific bio-receptor concentration for two hours. To improve the aptamer attachment to the gold surface, NaCl at the final concentration of $150\;\upmu {\text{M}}$ should be also added to the chamber and incubated for ten minutes. This provides an ionic force for salting out the oligonucleotides, that improves the aptamer attachment on the gold surface. A coverage of the florescent-tagged aptamers ($C_{1} = 1\;\upmu {\text{M}}$) on the surfaces of the MEMS cantilevers is illustrated in Fig. 5. As can be seen, the aptamers have been appropriately immobilized on the Au-coated sides of the cantilevers because of Au–S bond, while the density of these recognition elements is negligible on the other silicon sides. This coating causes a differential surface stress on the cantilevers to be effectively deflected up or down. Furthermore, it should be mentioned that the selection buffer used for the best interaction between aptamer and digoxin was prepared according to the literature^31^ which consists of $100\;{\text{mM}}\;{\text{NaCl}}$, $20\;{\text{mM}}\;{\text{Tris}}{-}{\text{HCl}}$ $\left( {pH 7.6} \right),$ $2\;{\text{mM}}$ ${\text{MgCl}}2, 5\;{\text{mM}}\;{\text{KCl}}$, $1\;{\text{mM}}\;{\text{CaCl}}_{2}$ and $0.02\% Tween 20$. In addition, the elution buffer^31^, which was used to dissociate aptamer and digoxin interactions, includes $40\;{\text{mM}}\;{\text{Tris}}{-}{\text{HCl}}$, $10\;{\text{mM}}\;EDTA$, $3.5\;{\text{M}}\;urea$, and $0.02\% Tween 20$ with $pH 8$.

#### Optical read-out

The light source employed in the proposed optical readout is a red laser diode with a wavelength of 638 nm and a power of 100mW. Additionally, Nikon D3400 has been used as the CCD camera, specified by a 24.2 MP APS-C CMOS sensor with a high image resolution of 6000 × 4000 (24.0 MP, 3:2), and a lens of 18-55 mm f/3.5–5.6G VR. Before performing the experiments, optical setup should be fixed in an optical table to eliminate any unwanted displacements. In this setup, the optical elements have been assembled as an optical interferometric microscope to monitor the data. In addition, the BioMEMS cantilever is fixed on its designed carrier, anchored in a particular position under the objective lens as shown in Fig. 5. In each experiment of this case study, $n = 20$ images of the biosensing platform have been quickly recorded for each concentration. All images are automatically captured by the related software to remove any unwanted errors in the setup. The cantilever zero-position images captured in a buffer injection (without digoxin) is used as reference data. It should be noted that capturing several images per concentration as explained in the previous Section (i.e., $n = 20$ images in this scenario) is for the use in the proposed image processing algorithm to minimize noises.

#### Image processing

Final results have been extracted by the proposed image processing algorithm through MATLAB software. This algorithm briefly shown in Fig. 4 and fully described in the previous section is based on the analysis of the shifts occurred in the red pixel intensity profile caused by the cantilever displacement. As a consequence, the output results of the proposed algorithm can be plotted as the biosensor calibration curve which is used to obtain the functional characteristics of the proposed MOM bio-platform, explained in the next section.

## Experimental results

In this sub-section, experimental results obtained from the MOM bio-platform is presented for the digoxin case study. Three levels of digoxin concentrations are identified by the functionalized platforms which are corresponding to different packing densities of aptamers coated on the cantilever surface $(C_{P0} = 0.5\;\upmu {\text{M}},\;C_{P1} = 1\;\upmu {\text{M}}$ and $C_{P2} = 3\;\upmu {\text{M}}).$ These levels have been considered in various ranges of $\left( {400\;{\text{pM}} - 10\;{\text{nM}}} \right),$ $\left( {2\;{\text{pM}} - 100\;{\text{pM}}} \right)$ and $\left( {50\;{\text{fM}} - 5\;{\text{pM}}} \right).$

**Figure 6:**
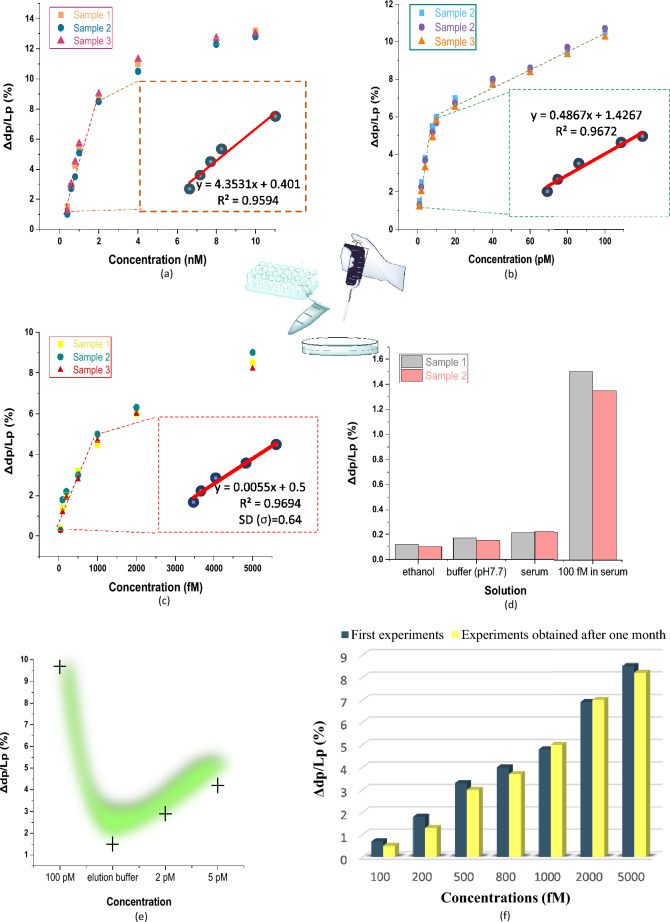
The experimental results of present MOM biosensor for identification and measurement of digoxin concentrations, (**a**) the percentage of displacement changes in the red pixel intensity profile versus various digoxin concentrations in the range of $400\;{\text{pM}}$–$10\;{\text{nM}}$ for therapeutic drug monitoring, (**b**) the percentage of displacement changes in the red pixel intensity profile versus various digoxin concentrations in the range of $1\;{\text{pM}}$–$100\;{\text{pM}}$, (**c**) the percentage of displacement changes in the red pixel intensity profile versus various digoxin concentrations in the range of $50\;{\text{fM}}$–$5\;{\text{pM}}$ dissolved in the plasma-like buffer sample for determining the detection limit of the BioMEMS biosensor, (**d**) the percentage of displacement changes in the red pixel intensity profile in the cantilever response to solutions with different impurity, (**e**) the percentage of displacement changes in the red pixel intensity profile per various digoxin concentrations before and after an elution buffer $\left( {pH 8.7} \right)$ injection, (**f**) the biosensor stability study by comparing the first test results for the proposed BioMEMS platform and the results obtained after one month (the percentage of displacement changes in the red pixel intensity profile versus various digoxin concentrations).

Digoxin concentrations in the range of $400\;{\text{pM}}$–$10\;{\text{nM}},$ diluted by a selection buffer $\left( {pH 7.7} \right),$ have been firstly analyzed by the BioMEMS platform to verify its precision in the digoxin therapeutic range ($0.5 - 0.9\;{\text{ng}}/{\text{mL}}$ or $0.64 - 1.15\;{\text{nM}}$ for adults^33^). The result of each concentration is obtained after eight minutes (the response time). As can be seen in Fig. 6a, the sensor can be appropriately applied to detect the digoxin concentrations in the examined range with a detection sensitivity of $S_{o} = 4.35 \times 10^{9} \;{\text{AU}}/{\text{M}}.$ Therefore, this highly sensitive and fast-response sensing structure is a promising choice in therapeutic drug monitoring for Point-Of-Care-Testing (POCT) applications in order to determine the toxic dosages of medications by regular measurement of drug concentrations in patient samples.

In addition, two other concentrations of bio-receptors have been immobilized on the sensor surface not only to show the ability of the biosensor operation in monitoring different ranges of biomarker concentrations, but also to optimize its detection limit. In $C_{p1} = 1\;\upmu {\text{M}},$ the digoxin concentrations in the range of $2\;{\text{pM}}$–$100\;{\text{pM}}$ have been precisely measured in two distinct linear regions with a maximum sensitivity of $S_{1} = 4.23 \times 10^{11} \;{\text{AU}}/{\text{M}}.$ This sensitivity is resulted from the linear approximation of data in the range of $2 - 10\;{\text{pM}}$, as illustrated in Fig. 6b.

On the other hand, other cantilever wafers have been prepared by the packing densities of $C_{p2} = 3\;\upmu {\text{M}}$ to provide a better coverage of the desired bioreceptors on the cantilevers surfaces and thus enhance the functional characteristics of the proposed biosensor. This is due to the fact that a surface stress-based operation is completely dependent to the number and the connectivity of binding events in recognition elements which determine the amount of released energies on the surface. As a result, for the devices functionalized by this packing density of anti-digoxin aptamers, the experiments have been carried out in selection buffer and plasma-like buffer samples with a drug concentration range of $50\;{\text{fM}} - 5\;{\text{pM}}.$ In fact, the plasma-like buffer containing bovine serum albumin (BSA) is used in order to analyze the effect of protein interferences in the performance of the implemented biosensing platform (the measurement precision). As can be seen in Fig. 6c, the slope of the calibration curve in the specified range results in the sensitivity of the BioMEMS platform, obtained as $S_{3} = 5.5 \times 10^{12} \;{\text{AU}}/{\text{M}}$. Furthermore, the limit of detection (LOD) of the implemented platform, as one of the most important functional characteristics of a biosensor, is calculated for this case study by^33^:

$$LOD = \frac{3.3\sigma }{S}$$

where σ is standard deviation of y-intercept and S is the sensitivity of the biosensor. Accordingly, the optimized device represents a detection limit of $LOD = 300 \times 10^{ - 15} \;{\text{M}}$ for the identification of digoxin concentrations which is remarkable in label-free biosensing.

Note that the selectivity of the device can be also determined by the changes analysis in the sensor response to different solutions including ethanol, buffer and plasma-like buffer sample (in the absence of target molecules). This can be shown in Fig. 6d for two samples where the effect of increasing impurity in sample solutions is negligible on the mechanical response of the cantilevers. In addition, an elution buffer $\left( {pH 8.7} \right)$ is used to evaluate the ability of disrupting or reversing the binding between the aptamers and digoxin with no damage in bio-receptors. The results are represented in Fig. 6e where the response of the cantilevers to pH changes is studied. Based on the downward deflection of the MEMS transducers in digoxin binding events, there is an upward reaction in the cantilevers after injection of the elution buffer. This shows the buffer efficiency to reverse the biological interactions. Moreover, due to the regeneration of ligand-analyte binding, which leads to the proper deflection of the cantilevers in two different concentrations, one can conclude that this pH changes cannot affect the performance of DNA aptamers.

Finally, the stability of the implemented biosensor has been studied over one month. For this purpose, a functionalized platform has been saved in a fixed condition (a temperature of 4 °C) to be tested one month later. As represented in Fig. 6f, the experimental results are appropriately close to each other so that the initial experiments can confirm the second ones and vice versa.

## Discussion

Currently, mass spectrometry and chromatography are the most common approaches for detection of tiny-molecule drugs in blood samples^34^. Considering the importance of digoxin medicine in treatment of heart diseases and its narrow therapeutic index, the development of label-free detection of digoxin is important for rapid and cost-effective therapeutic drug monitoring in POCT applications. However, this issue is slightly addressed in recent contributions^34–39^. Few immuno-based assays^34^, electrochemical probes^35,36^, nanobiosensor based on localized surface plasmon resonances^37^ and fluorescent biosensors^38–40^ have been reported for the digoxin analysis with the detection ranges from 8 Picomolar to few Nanomolar concentrations, as summarized in Table 1. As a consequence, the proposed optical BioMEMS device is a label-free detection method which overcomes the other reported approaches for measuring digoxin concentrations in plasma samples with a very low limit of detection as $LOD = 300\;{\text{fM}}$ and a sensitivity of $S = 5.5 \times 10^{12} \;{\text{AU}}/{\text{M}}.$

**Table 1:** The operation of the proposed biosensor compared to several recent approaches for digoxin identification.

References	Operating mechanism	Measurement sample	Detection limit (pM)
^35^	Electrochemical modified by Au-NPs-GO-	PBS buffer	115.2
^36^	AuNP Surface Enhanced Raman Scattering (SERS-ELISA)	PBS buffer	512
^37^	LSPR	PBS buffer and FBS	2560
^38^	Fluorescent based on silica NPs	Rat serum	566
^39^	Fluorescent using reduced graphene quantum dots	Human serum	8
^40^	Fluorescent based on AuNPs and g-C3N4NS	Human plasma	4.1
The proposed platform	Optical MEMS	Plasma-like buffer	0.3

On the other hand, according to the literature^17,41,42^, mechanical biosensors relied on surface stress generation represent different limits of detection from $500\;{\text{fM}}$ to nanomolar ranges for various applications. It should be mentioned that in most surface stress-based biosensors relying on optical detection systems, the reflection of the illuminated light from the cantilever surface are monitored by position sensitive detectors (PSD) to measure any deflection changes. Thus, the PSD-based methods require reference cantilevers and calibration approaches for precisely determining deflections, because they are highly susceptible to the intensity noise of light sources, contrarily to the proposed BioMEMS platform. Furthermore, the implementation of these surface stress-based cantilever structures is more complicated compared to the present proposed approach, due to the geometrical constraints of optical detections (i.e., the necessity to provide cantilevers angled away from each other).

Therefore, our biological system can be used as an ultra-sensitive label-free biosensor to precisely detect a specific biomarker or multiple biomarkers in disease diagnosis by the targeted immobilization of various desired bioreceptors on its bio-transducer platform. Consequently, the proposed highly-sensitive MOM bio-platform can be a promising choice for the next generation of commercialized label-free biosensors in POCT applications.

## Conclusion

In this research work, we proposed a multipurpose Optical MEMS (MOM) bio-platform for rapid and cost-effective therapeutic drug monitoring and early diagnosis in POC applications. We experimentally show that our platform can be used for various measurement ranges of biomarker concentrations (ranges of nM, pM or even fM and aM) which is significant for detection of various types of bio-molecules. This can be provided by controlling the packing density of bioreceptors in the SAM functionalization procedure. An experimental implementation of the proposed platform is then carried out for identification and measurement of digoxin. The measured results show that the present BioMEMS device can be a fully-integrated, comprehensive platform for diagnosis of diseases with very low concentrations of biomarkers in target samples. This is realized by the use of the proposed high resolution optical detection approach relied on wave interference phenomenon which can record tiny nanomechanical changes of the sensitive cantilevers.

As an on-going work, we aim to produce a minimum viable product of the proposed bio-platform as a comprehensive portable device. This can be connected to a digital equipment (such as a smartphone, a computer) for data processing and monitoring the results. The total solution including a fluidic channel, the optical detection system (i.e. a laser diode, a CCD sensor) and the functionalized bio-transducer can be compacted in a small package in order to transfer measured signals (after a few minutes) to a simple processing unit for image processing and finally to obtain the output results. Note that, since the bio-transducer platform can be functionalized and characterized by various recognition elements related to a specific application, this system can be also used as a multipurpose biomedical tool in biosensing applications. Thus, a cost-effective biosensor can be manufactured for personal homecare diagnostics which can precisely measure very low concentrations of small biomolecules in therapeutic drug monitoring or disease diagnosis to prevent the progression of chronic diseases (i.e., cancers, heart diseases or neurodegenerative disorders).

## Data Availability

The datasets used and/or analyzed during the current study available from the corresponding author on reasonable request.
